# Supplementary material for: Gated recurrence enables simple and accurate sequence prediction in stochastic, changing, and structured environments
Source: eLife. 2021 Dec 2;10:e71801. doi: 10.7554/eLife.71801 (PMC8735865; doi:10.7554/eLife.71801)
Supplement: Transparent reporting form [file elife-71801-transrepform1.docx]

***eLife’s* transparent reporting form**

We encourage authors to provide detailed information *within their submission* to facilitate the interpretation and replication of experiments. Authors can upload supporting documentation to indicate the use of appropriate reporting guidelines for health-related research (see [EQUATOR Network](http://www.equator-network.org/ )), life science research (see the [BioSharing Information Resource](https://biosharing.org/)), or the [ARRIVE guidelines](http://www.plosbiology.org/article/info:doi/10.1371/journal.pbio.1000412) for reporting work involving animal research. Where applicable, authors should refer to any relevant reporting standards documents in this form.

If you have any questions, please consult our Journal Policies and/or contact us: [editorial@elifesciences.org](mailto:editorial@elifesciences.org).

**Sample-size estimation**

- You should state whether an appropriate sample size was computed when the study was being designed
- You should state the statistical method of sample size computation and any required assumptions
- If no explicit power analysis was used, you should describe how you decided what sample (replicate) size (number) to use

Please outline where this information can be found within the submission (e.g., sections or figure legends), or explain why this information doesn’t apply to your submission:

We have not done explicit power analysis. We used n=20 agents of each network and heuristic type to test for reproducibility. We used this sample size (n=20) because it is similar to the typical sample size used in the experimental studies that we model (Behrens et al Nat Neuro 2007; Meyniel et al PCB 2015; Meyniel et al PNAS 2017; Heilbron et al 2020) and it is comparable to, if not larger than, related theoretical studies (e.g. Wang et al Nat Neuro 2018 used n=8 in Fig 3) indicating that n=20 should provide sufficient statistical power.

**Replicates**

- You should report how often each experiment was performed
- You should include a definition of biological versus technical replication
- The data obtained should be provided and sufficient information should be provided to indicate the number of independent biological and/or technical replicates
- If you encountered any outliers, you should describe how these were handled
- Criteria for exclusion/inclusion of data should be clearly stated
- High-throughput sequence data should be uploaded before submission, with a private link for reviewers provided (these are available from both GEO and ArrayExpress)

Please outline where this information can be found within the submission (e.g., sections or figure legends), or explain why this information doesn’t apply to your submission:

The sample-size is reported in the Method/Training section. Each experiment with n=20 agents was performed only once (but the code that we provide enables the reader to replicate the results with more experiments). The number of sequences and minibatches used for each experiment is reported in the Method/Training section.

No data was excluded from the analysis.

**Statistical reporting**

- Statistical analysis methods should be described and justified
- Raw data should be presented in figures whenever informative to do so (typically when N per group is less than 10)
- For each experiment, you should identify the statistical tests used, exact values of N, definitions of center, methods of multiple test correction, and dispersion and precision measures (e.g., mean, median, SD, SEM, confidence intervals; and, for the major substantive results, a measure of effect size (e.g., Pearson's r, Cohen's d)
- Report exact p-values wherever possible alongside the summary statistics and 95% confidence intervals. These should be reported for all key questions and not only when the p-value is less than 0.05.

Please outline where this information can be found within the submission (e.g., sections or figure legends), or explain why this information doesn’t apply to your submission:

Statistical methods are described in Method/Statistics.

Raw data (with dots for the 20 agents) are shown on most figures; expect on Fig 3b, 6c, 7 that explore a range of conditions and where reporting raw data would have resulted in a very cluttered plot (we report 95% confidence intervals in that case).
We report effect sizes, confidence intervals and significance levels.

(For large datasets, or papers with a very large number of statistical tests, you may upload a single table file with tests, Ns, etc., with reference to sections in the manuscript.)

**Group allocation**

- Indicate how samples were allocated into experimental groups (in the case of clinical studies, please specify allocation to treatment method); if randomization was used, please also state if restricted randomization was applied
- Indicate if masking was used during group allocation, data collection and/or data analysis

Please outline where this information can be found within the submission (e.g., sections or figure legends), or explain why this information doesn’t apply to your submission:

Not applicable (no group allocation here).

**Additional data files (“source data”)**

- We encourage you to upload relevant additional data files, such as numerical data that are represented as a graph in a figure, or as a summary table
- Where provided, these should be in the most useful format, and they can be uploaded as “Source data” files linked to a main figure or table
- Include model definition files including the full list of parameters used
- Include code used for data analysis (e.g., R, MatLab)
- Avoid stating that data files are “available upon request”

Please indicate the figures or tables for which source data files have been provided:

All plots and related data can be reproduced exactly with the code that we provide.

For now, the code is available on a private URL (see figshare link in the Method/Code availability section), which we will turn public upon publication.

We provide in Table #1 the hyperparameters corresponding to the optimization of the networks.
